# Phenotypic Changes Associated With *In Vivo* Evolution of Colistin Resistance in ST11 Carbapenem-Resistant *Klebsiella pneumoniae*

**DOI:** 10.3389/fcimb.2022.841748

**Published:** 2022-02-24

**Authors:** Miaomiao Xie, Kaichao Chen, Ning Dong, Qi Xu, Edward Wai-Chi Chan, Rong Zhang, Sheng Chen

**Affiliations:** ^1^ Department of Infectious Diseases and Public Health, Jockey Club College of Veterinary Medicine and Life Sciences, City University of Hong Kong, Hong Kong, Hong Kong SAR, China; ^2^ Department of Medical Microbiology, School of Biology and Basic Medical Science, Medical College of Soochow University, Suzhou, China; ^3^ State Key Lab of Chemical Biology and Drug Discovery, Department of Applied Biology and Chemical Technology, The Hong Kong Polytechnic University, Hong Kong, Hong Kong SAR, China; ^4^ Department of Clinical Laboratory, School of Medicine, Second Affiliated Hospital of Zhejiang University, Hangzhou, China

**Keywords:** *Klebsiella pneumoniae*, colistin resistance, *in vivo* evolution, fitness, virulence

## Abstract

Colistin is one of the few antibiotics that exhibit bactericidal effect on carbapenemase-producing *Klebsiella pneumoniae* strains. In recent years, however, colistin resistance is increasingly being reported among clinical carbapenem-resistant *K. pneumoniae* strains worldwide, posing serious challenge to treatment of infections caused by these organisms. In this study, we investigated one colistin-susceptible (YJH4) and one colistin-resistant (YJH15) *K. pneumoniae* strain, which were collected from a patient before and after colistin treatment, respectively. We characterized the effects of *mgrB* inactivation-induced colistin resistance on the physiological fitness and virulence in ST11 carbapenem-resistant *K. pneumoniae* both *in vitro* and *in vivo*. The colistin-resistant strain YJH15 was found to exhibit increased fitness and biofilm formation potential *in vitro*, and increased survival rate in the presence of normal human serum. Interestingly, YJH15 exhibited reduced virulence in the mouse infection model but enhanced virulence in *Galleria mellonella* infection model when compared to the colistin-susceptible parental strain YJH4. Infection with YJH15 was also found to result in lower expression level of inflammatory cytokine IL-1β in blood and significantly decreased bacterial loads in heart, liver, spleen, lung, kidney and blood. These results demonstrated that *mgrB* inactivation-induced colistin resistance has significant effects on multiple fitness and virulence-associated traits in *K. pneumoniae*.

## Introduction

*Klebsiella pneumoniae* is one of the most common Gram-negative pathogens that cause nosocomial infections, particularly among patients suffering from severe illnesses (Gomez-Simmonds and Uhlemann, 2017). The incidence of carbapenem-resistant *K. pneumoniae* (CRKP) has significantly increased worldwide in recent decades (Munoz-Price et al., 2013; Hu et al., 2016). Polymyxins such as colistin has become the only therapeutic option effective for treatment of infections caused by these carbapenemase-producing organisms before the introduction of ceftazidime-avibactam, meropenem-vaborbactam and imipenem-relebactam in the clinical practice (Tumbarello et al., 2012). However, increased usage of colistin in agriculture and healthcare settings has resulted in the emergence and rapid dissemination of colistin resistant, carbapenemase-producing *K. pneumoniae* strains (Ah et al., 2014). Colistin resistance is mainly caused by modification of antibiotic target as a result of chromosomal mutations in gene *pmrAB*, *phoPQ* and *mgrB* or acquisition of exogenous genes such as *mcr-1*, which acts by upregulating expression of the lipopolysaccharide modification enzymes. Structural alterations in two-component regulatory systems including PmrAB and PhoPQ, as well as mutations in the *mgrB* gene, are the main causes of colistin resistance in *K. pneumoniae* (Cannatelli et al., 2014). Inactivation of *mgrB* gene which encodes a negative regulator of PhoPQ two-component system is a particularly common mechanism of colistin resistance in *K. pneumoniae*. The inactivation effect may be due to insertion of an insertion sequence or a mutation that results in generation of a premature stop codon (Cannatelli et al., 2014). In addition, plasmid-mediated colistin resistance genes including the *mcr-1* gene, which have been reported in recent years, can also confer colistin resistance in members of Enterobacteriaceae (Liu et al., 2016; Xavier et al., 2016). The relationship between colistin resistance and fitness cost, as well as virulence, have been reported in some Gram-negative pathogens such as *K. pneumoniae* and *Acinetobacter baumannii*. A previous study showed that the *mgrB* gene mutation induces PhoPQ-mediated lipid A remodeling, which in turn confers colistin resistance and enhanced virulence in *K. pneumoniae* through increasing resistance to antimicrobial peptide and impairing activation of host defense response (Kidd et al., 2017). Furthermore, colistin resistance is known to be associated with a significant biological cost in terms of overall physiological fitness and virulence in *A. baumannii* (Beceiro et al., 2014).

In this study, we characterized the effects of *mgrB* inactivation-induced colistin resistance on the physiological fitness and virulence in ST11 carbapenem-resistant *K. pneumoniae* both *in vitro* and *in vivo*. The expression levels of cytokines in a mouse infection model, as well as biofilm production and serum resistance, were examined in two *K. pneumoniae* strains sequentially collected from a clinical treatment with *in vivo* natural evolution of colistin resistance. In addition, the physiological fitness and changes in virulence related phenotypes during the course of evolution of colistin resistance in ST11 carbapenem-resistant *K. pneumoniae* were determined by performing *in vitro* and *in vivo* competition experiments, as well as assessing the virulence level of the test strains in a mouse sepsis model.

## Materials and Methods

### Bacterial Strains and Antimicrobial Susceptibility Tests

ST11 carbapenem-resistant *K. pneumoniae* strain YJH4 was isolated from a blood sample of a 29-years-old male patient diagnosed with acute monocytic leukemia. The patient received chemotherapy and antimicrobial treatment with meropenem and vancomycin (Zhang et al., 2018). Strain YJH4 was isolated initially from a blood sample after the patient underwent hematopoietic stem cell transplantation in 2015. *K. pneumoniae* YJH15 was collected from a fecal sample in 2017 after the patient gradually recovered and remained healthy since then. Strain YJH15 was found to be resistant to colistin and enhanced minimal inhibitory concentration (MIC) to tigecycline (**Table 1**). Phylogenetic analysis of the two *K. pneumoniae* strains indicated that they belonged to the identical genetic clone with less than 1.5% genetic difference. Single nucleotide polymorphisms (SNP) analysis revealed that the number of SNPs between these two isolates was 8, verifying that they belonged to an identical genetic clone. Genome analysis showed that an insertion sequence IS*903B* was located between the 13^th^ and 14^th^ base pair of *mgrB* in strain YJH15 when compared to strain YJH4, suggesting that continual usage of colistin in the patient caused the evolution and selection of colistin resistance *in vivo* through inactivation of *mgrB* gene. In addition, development of tigecycline resistance was attributed to the induction of *tet(A)* gene expression in the tigecycline-sensitive strain YJH4 that contain a *tet(A)* variant upon treatment with tigecycline for a prolonged period. The colistin and tigecycline resistant CRKP strain YJH15 could not be eradicated by colistin treatment, and persisted in the gastrointestinal tract of the patient for a long period of time even in the absence of antibiotic selection pressure. Minimal inhibitory concentrations (MIC) of these two test strains were determined using broth microdilution method according to the Clinical and Laboratory Standards Institute (CLSI) (CLSI, 2018) guidelines. Antimicrobial susceptibility was defined based on the CLSI breakpoints.

**Table 1:** Phenotypic characteristics of *K. pneumoniae* strains YJH4 and YJH15.

Strain ID	Source	Isolation date	*bla*_KPC-2_	MLST	MIC (µg mL ^-1^)								
					MEM	FEP	FOS	AMK	LEV	CST	SCF	TIG	ISE
YJH4	Blood	11/05/15	+	ST11	>32	>256	>256	>256	256	0.25	>256	1	>128
YJH15	Feces	12/07/17	+	ST11	32	>256	>256	>256	>256	64	256	4	>128

MEM, meropenem; FEP, cefepime; FOS, fosfomycin; AMK, amikacin; LEV, levofloxacin; CST, colistin; SCF, cefperazone/sulbactam; TIG, tigecycline; ISE, isepamicin.

### *In Vitro* Growth

To construct an *in vitro* growth curve, *K. pneumoniae* strains were grown in both Lysogeny Broth (LB) and human serum. To assess the relative *in vitro* fitness under noncompetitive environment, growth rate in LB was recorded. Briefly, *K. pneumoniae* strains were grown in LB overnight at 37°C with constant shaking. Bacterial culture was then adjusted to OD600 of 0.1 and 1000-fold diluted in 5mL LB, followed by incubating at 37°C with shaking (250rpm). Viable cells were determined by spreading serial dilutions on LB agar plates at 0, 1, 2, 3, 4, 5, 6, 7, 8, 9, 10, 11, 12 and 24 h post-dilution (Beceiro et al., 2014). The growth of *K. pneumoniae* strain in human serum was determined as previously described (Siu et al., 2011). Briefly, bacterial suspension containing 2*10^6^ CFU mL^-1^ was collected from exponential phase cultures, followed by mixing with pooled human serum (Sigma-Aldrich) at a ratio of 1:3 and incubated at 37°C with constant shaking. CFU counts were determined for the initial mixture as well as that after 1, 2, and 3hrs of incubation. Serum resistance was delineated by depicting the survival percentage of each strain over time. An bacterial strain was regarded as susceptible to serum if colony counts dropped to 1% of initial inoculum, and resistant if more than 90% organisms remained alive after 2hrs of incubation (Jadhav et al., 2011).

### Biofilm Formation

Biofilm formation potential of test strains was tested as previously described with slight modifications (Wu et al., 2011). Briefly, *K. pneumoniae* strain was incubated in Mueller Hinton (MH) broth. Overnight bacterial culture was then adjusted to 0.5 McFarland turbidity standards and diluted 100-fold with fresh MH broth. 200µl of bacterial culture were incubated into wells of 96-well flat-bottomed polystyrene plates for 24h at 37°C. Five replicates were conducted for each strain and wells containing only MH broth were included as negative control. Bacterial culture was aspirated and rinsed four times with phosphate buffered saline (PBS) to remove planktonic bacteria. Biofilm produced by bacteria that adhered firmly to the wells was heat-fixed by incubating at 60°C for 3 hours, followed by staining with 0.5% crystal violet for 15min. Excess stain was rinsed off and the plates were dried in room temperature. To assess the amount of biofilm formed, crystal violet bound to the adherent bacteria was dissolved with 200µl 33% acetic acid solution and optical density was recorded at 595nm after incubation for 10 minutes.

### Cell Culture and Killing Assay

Human leukemic cell line (HL-60) was maintained in RPMI 1640 growth medium supplemented with 20% fetal bovine serum at 37°C. For differentiation into neutrophil-like cells, HL-60 cells were cultured in the presence of 1.25% dimethyl sulfoxide for three days. The dHL-60 cells (5*10^5^ cells) were then challenged with *K. pneumoniae* strain at a multiplicity of infection of 1:100 and incubated at 37°C for 1h, 2h and 3h in antibiotic-free RPMI medium in a 24-well plate. To sufficiently synchronize infection, the plates were centrifuged at 200*g for 5min. After incubation, survival rate of test strain was determined by spreading serial dilutions onto LB agar plates and incubated at 37°C. The survival rate was depicted as the percentage of *K. pneumoniae* population that survived after treatment with dHL-60 cells.

### *In Vitro* and *In Vivo* Competition Assay

To compare the relative fitness of colistin-resistant YJH15 with that of colistin-susceptible parental strain YJH4, *in vitro* competitive index (CI) was determined as previously described (Yang et al., 2017). Briefly, bacterial suspensions of the two strains were collected from cultures in logarithmic phase and mixed at a ratio of 1:1 (approximately 1.5*10^3^ CFU). The mixture was added into 10mL M9 medium supplemented with 0.4% glucose, followed by incubation at 37°C with shaking (250rpm) for 18h. Total CFU in the mixture as well as the proportion of colistin-resistant isolate YJH15 in the mixture was determined by spreading serial dilutions on LB agar plates and LB agar plates containing 8µg mL^-1^ colistin. Competitive index was described as a ratio of CFU of colistin-resistant strain to colistin-susceptible parental strain. By definition, a competitive index of 1 represents no fitness cost, whereas a competitive index higher or lower than 1 represents enhanced or decreased fitness, respectively. To investigate *in vivo* competition of these two strains, mice (n = 4) were infected with *K. pneumoniae* YJH4 and YJH15 (1*10^6^ CFU of each isolate from logarithmic phase mixed at a ratio of 1:1) by intravenous inoculation. At 24h post-infection, mice were sacrificed and organ homogenates including that of heart, lung, liver, spleen, kidney and blood were collected, serially diluted in PBS, and spread onto LB agar plates and LB agar plates containing 8µg mL^-1^ colistin, recording the number of colonies that appeared after overnight incubation at 37°C.

### Virulence Potential of Test Strains in *Galleria mellonella* and Mouse Infection Model

The discrepancy between the virulence level of *K. pneumoniae* YJH4 and YJH15 was evaluated in both *G. mellonella* and mouse infection model. *G. mellonella* weighing approximately 300mg were surface-sterilized with 75% ethanol and subjected to infection by 1*10^4^ CFU of *K. pneumoniae* strain (n=10 larvae per group), using a Hamilton syringe as described previously (McLaughlin et al., 2014). The larvae were then placed in petri dishes and incubated at 37°C. Survival of *G. mellonella* was recorded for 48h post-infection. Experiments were allowed to proceed for three days at the most since pupa formation may occur on the fourth day. For the mouse infection model, ICR mice (4 weeks-old, average 25g) were used. Ten mice randomly allocated to each group were infected with 2.5*10^7^ CFU of *K. pneumoniae* by intravenous injection. Survival of mice in each group was recorded at 12h intervals for a total of 120hrs post-infection. Animal infection experiments were conducted twice to confirm data consistency. Survival curves were constructed by GraphPad Prism 8 and statistical analysis performed by the Log-rank (Mantel-Cox) test.

### Quantitative Real-Time PCR Analysis of Blood Cytokines in Mouse Infection Model

Four mice randomly allocated in each group were infected with 1.0*10^6^ CFU of *K. pneumoniae* YJH4 or YJH15 by intravenous injection, respectively. Mice in the control group were inoculated with sterile normal saline. Blood samples were collected in each group by cardiac puncture 24hrs post-infection. Total RNA in blood was extracted and purified using the RNeasy kit (QIAGEN) and Turbo DNA free kit (Invitrogen) according to the manufacturer’s instructions. Reverse transcription analysis was conducted using SuperScript III quantitative one-step kit (Invitrogen), with GAPDH being used to normalize the expression levels of test genes. Primers used were listed in **Table 2**. Quantitative real-time PCR analysis was undertaken by using the QuantStudio 5 Real-Time PCR System (Applied Biosystems) and SYBR Green master mix (Applied Biosystems). Results were analyzed by QuantStudio™ Design and Analysis Software v1.5.1.

**Table 2:** Primers used in quantitative real-time PCR analysis.

Primer	Sequence (5’ to 3’)	Source
GAPDH FW	AGGTCGGTGTGAACGGATTTG	This study
GAPDH RV	TGTAGACCATGTAGTTGAGGTCA	This study
IL-1β FW	CTTCAGGCAGGCAGTATCACTC	This study
IL-1β RV	TGCAGTTGTCTAATGGGAACGT	This study
IL-6 FW	ACAACCACGGCCTTCCCTAC	This study
IL-6 RV	TCTCATTTCCACGATTTCCCAG	This study
IL-10 FW	GCTCTTACTGACTGGCATGAG	This study
IL-10 RV	CGCAGCTCTAGGAGCATGTG	This study
IL-12b FW	ACAGCACCAGCTTCTTCATCAG	This study
IL-12b RV	TCTTCAAAGGCTTCATCTGCAA	This study
IFN-γ FW	GCTTTGCAGCTCTTCCTCAT	This study
IFN-γ RV	GCAGGATTTTCATGTCACCA	This study
TNF-α FW	CGAGTGACAAGCCTGTAGCCC	This study
TNF-α RV	GTCTTTGAGATCCATGCCGTTG	This study

### Statistical Analyses

Data was presented as means ± standard deviations. Statistical analyses were conducted by performing the students’ *t* test. Survival analyses were undertaken by performing the log-rank (Mantel-Cox) test. All analyses were conducted by using the GraphPad Prism (version 8.0.1) software.

### Ethics Statement

Animal infection experiments were approved by the Research Animal Care and Use Committee of City University of Hong Kong.

## Results

### Effect of *mgrB* Inactivation-Induced Colistin Resistance on *In Vitro* Growth

**Figure 1:**
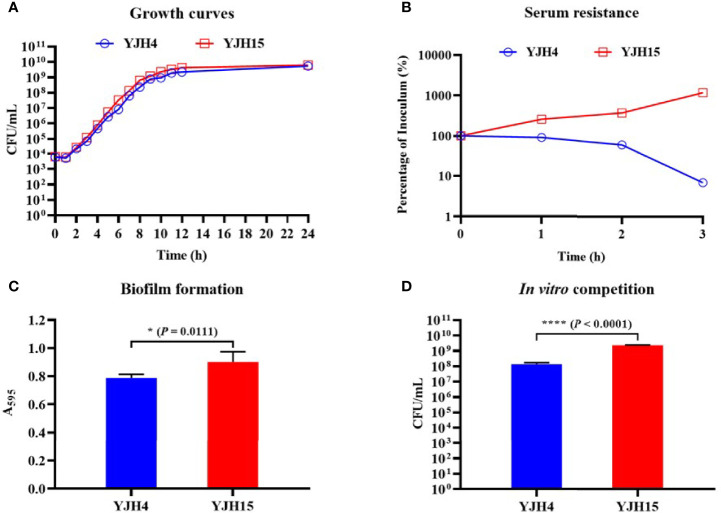
Effect of colistin resistance on *K*. *pneumoniae* phenotypic expression. **(A)** Growth curves of colistin-resistant and colistin-susceptible *K*. *pneumoniae* strains. *y* axis, viable bacterial cells (CFU/mL); *x* axis, hours of culture. **(B)** Serum resistance of colistin-susceptible parental strain YJH4 and colistin-resistant YJH15. Serum resistance is described as percentage of viability after 1, 2, and 3hrs of incubation (CFU at specified time/CFU of the initial mixture). **(C)** Biofilm formation of *K*. *pneumoniae* strains. Biofilm production was determined by detecting the absorbance at 595nm. Statistical analysis was conducted by performing Student’s *t* test. (*P = 0.0111). **(D)** *In vitro* competition assay in M9 medium. *K*. *pneumoniae* strains YJH4 and YJH15 were mixed at a ratio of 1:1 and grown together in M9 medium supplemented with 0.4% glucose. CFUs of strains YJH4 and YJH15 were determined after 18hrs’ co-culturation. (****P < 0.0001).

The effect of *mgrB* inactivation-induced colistin resistance on bacterial growth was assessed in both rich media (LB) and human serum. Although the colistin-resistant strain YJH15 grew slightly faster than its colistin-susceptible parental strain YJH4 (**Figure 1A**), growth rates of the colistin-resistant and colistin-susceptible strains in LB were similar. However, in human serum, colistin-resistant strain YJH15 exhibited a markedly increased growth rate while that of colistin-susceptible parental strain YJH4 decreased dramatically over the three-hour test period (**Figure 1B**), indicating that *K. pneumoniae* YJH15 was resistant to human serum upon development of resistance to colistin. The difference between growth rate recorded in rich medium and human serum indicates that exposure to an environment similar to those encountered by *K. pneumoniae* during human infection resulted in dramatic increase in the physiological fitness, prompting us to use *in vivo* models to mimic the process of *K. pneumoniae* infection.

### Effect of *mgrB* Inactivation-Induced Colistin Resistance on Biofilm Formation and Survival in HL-60 Cell Killing Assay

**Figure 2:**
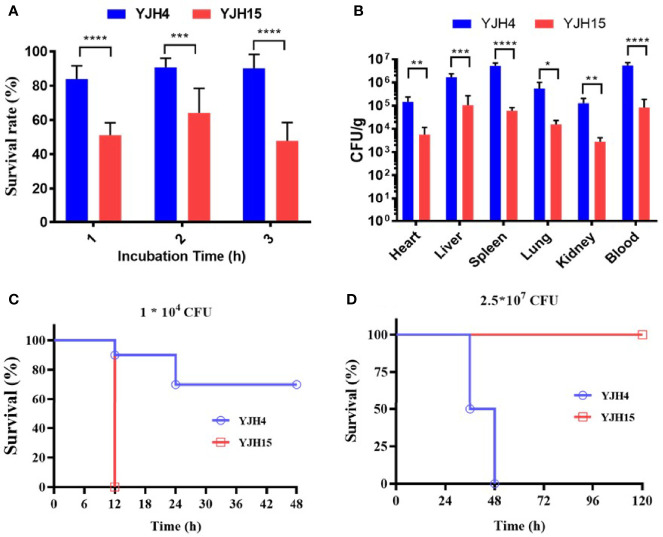
Effect of colistin resistance on *K*. *pneumoniae* virulence expression. **(A)** HL-60 cell killing assay. The survival rate was depicted as the percentage of *K*. *pneumoniae* strain that survived treatment with the dHL-60 cells. (***P < 0.001, ****P < 0.0001). **(B)** *In vivo* competition in mouse infection model. ICR mice were infected with a mixture of equal proportion of strain YJH4 and YJH15 (1*10^6^ CFU mixed at a ratio of 1:1). Bacterial loads of *K*. *pneumoniae* YJH4 and YJH15 in blood, heart, liver, spleen, lung and kidney homogenates of mice at 24hrs post-infection were determined. Data were presented as the mean of CFU g^-1^ ± SD and analyzed by unpaired *t* test. *~****, (*P* < 0.05 ~ *P* < 0.0001). **(C)** Virulence potential of colistin-resistant and colistin-susceptible *K*. *pneumoniae* in *G*. *mellonella* infection model. The percentage survival of *G mellonella* infected with 1*10^4^ CFU (****P < 0.0001) of each *K*. *pneumoniae* strain at 48hrs is shown (n = 10). **(D)** Virulence potential of colistin-susceptible YJH4 and colistin-resistant YJH15 in mouse infection model. The percentage survival of ICR mice infected with 2.5*10^7^ CFU of each *K*. *pneumoniae* strain at 120hrs is shown (**P = 0.0084). Ten mice were infected in each group. Statistical analysis of animal survival curves was conducted by performing the Log-rank (Mantel-Cox) test.

Using a polystyrene microtiter plate test approach, we examined the ability of *K. pneumoniae* strains to form biofilms. Strain YJH15 was found to exhibit a higher potential to form biofilm than the colistin-susceptible parental strain YJH4 (*P* = 0.0111) (**Figure 1C**). HL-60 cell killing assays were also performed, with results showing that colistin-resistant strain YJH15 exhibited much lower survival rates than that of YJH4 during three-hour co-culture with HL-60. Within the first hour, only 55.08% of strain YJH15 survived, but an 82.41% survival rate of YJH4 was recorded (*P*<0.0001). Consistently, 51.77% and 92.31% of YJH15 and YJH4 were found to survive after three hours’ exposure to HL-60 cell (*P*<0.0001), respectively (**Figure 2A**). These data suggested that survival fitness of colistin-resistant strain YJH15 was severely impaired.

### Fitness Cost of *mgrB* Inactivation-Induced Colistin Resistance Depicted by *In Vitro* and *In Vivo* Competition Assay

To examine the fitness costs of *mgrB* inactivation-induced colistin resistance in *K. pneumoniae*, competition assays were performed in both M9 medium supplemented with 0.4% glucose and mouse model by determining the number of colistin-resistant YJH15 and colistin-susceptible YJH4 cells that remained at the end of a competition assay, in which two strains were mixed and grown together. *In vitro* competition assay demonstrated that strain YJH15 exhibited a significant increase in fitness when co-cultured with parental strain YJH4 (CI value, 16.71; *P* < 0.0001) (**Figure 1D**). To determine the *in vivo* competition and systemic dissemination, ICR mice were inoculated with equal amounts of strain YJH4 and YJH15 (1*10^6^ CFU mixed at a ratio of 1:1) by intravenous inoculation. Bacterial loads in blood, heart, liver, spleen, lung and kidney homogenates were determined at 24hrs post-infection, with results showing that bacterial loads of colistin-resistant YJH15 decreased significantly in heart, liver, spleen, lung, kidney and blood (*P*<0.05 ~ *P*<0.0001) when compared with that of the colistin-susceptible parental strain YJH4 in those organs (**Figure 2B**), suggesting a significant decreased fitness *in vivo* after *K. pneumoniae* YJH15 has developed colistin resistance.

### Virulence Potential in *G. mellonella* and Mouse Infection Models

A strong correlation between the virulence potential of *K. pneumoniae* in *G. mellonella* and mouse infection models has been established (Jander et al., 2000). To evaluate the pathogenic potential of *K. pneumoniae* strains in *G. mellonella*, an equal amount of colistin-resistant strain YJH15 and colistin-susceptible strain YJH4 was injected into the worms. Infection of *G. mellonella* with 1*10^4^ CFU of colistin-resistant strain YJH15 resulted in 100% mortality at 12h post-infection, which was significantly higher than the mortality rate caused by colistin-susceptible strain YJH4, for which only 30% mortality was observed at 48h post-infection (*P* < 0.0001) (**Figure 2C**), suggesting that *mgrB* inactivation-induced colistin resistance caused a significant increase in virulence of *K. pneumoniae* in *G. mellonella*. The enhanced virulence potential of colistin-resistant *K. pneumoniae* YJH15 in *G. mellonella* infection model prompted us to evaluate the ability of this colistin resistant isolate to cause infection in a mouse model. Interestingly, mouse infection experiments produced a contradictory result. Infection of ICR mice with 2.5*10^7^ CFU of colistin-susceptible strain YJH4 resulted in 50% mortality at 36hrs post-infection and 100% mortality at 48hrs post-infection, which was significantly higher than the mortality rate recorded in infection by the colistin-resistant strain YJH15 (*P* = 0.0084), in which 0% mortality was recorded at 120hrs (**Figure 2D**). These findings suggested that colistin-resistant *K. pneumoniae* YJH15 exhibited decreased virulence in mouse infection model but increased virulence in *G. mellonella* infection model.

### Expression Level of Blood Cytokine in Mouse Infection Model

**Figure 3:**
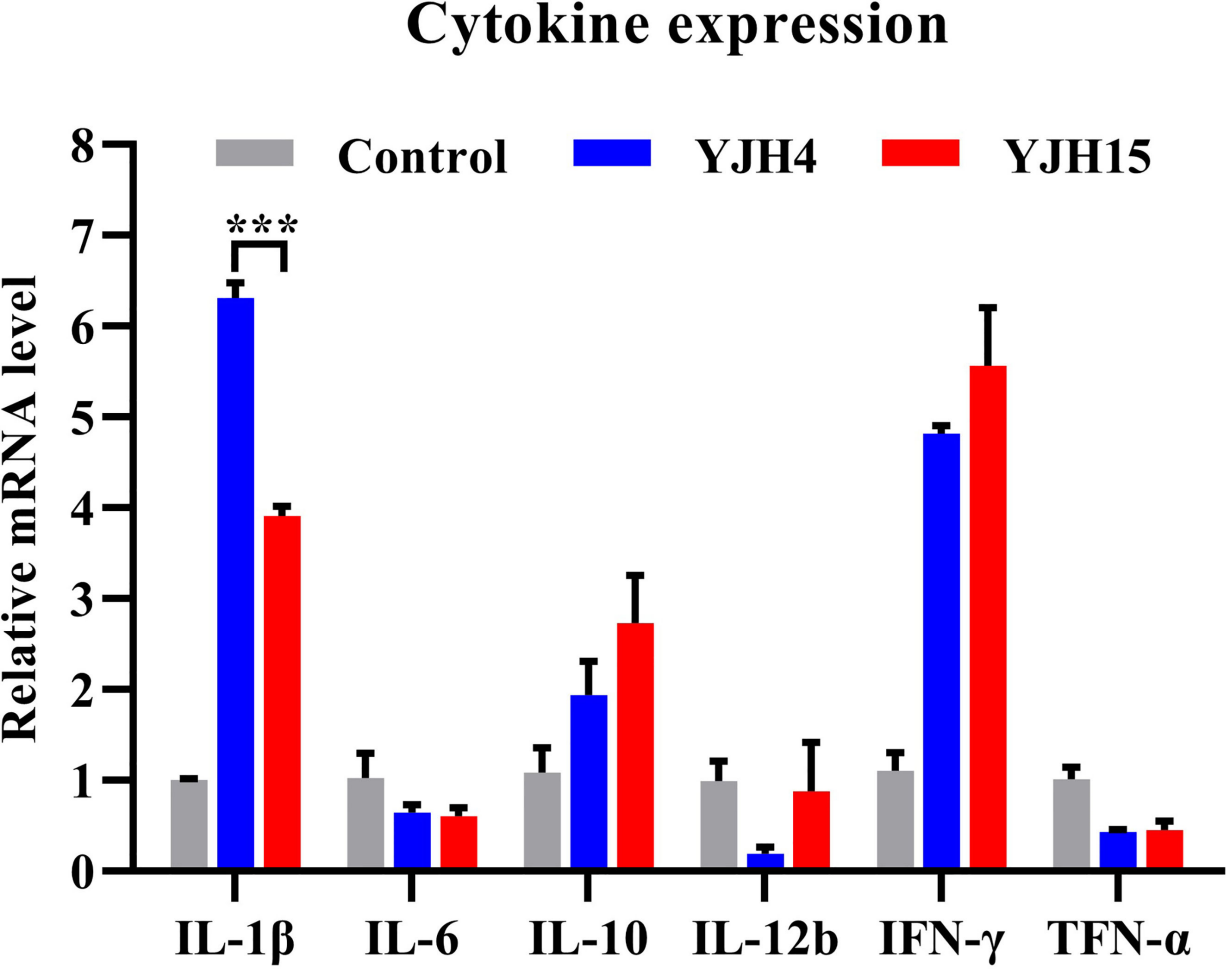
Expression levels of blood cytokine in mice infected with *K. pneumoniae* YJH4 and YJH15. Expression levels of cytokines produced in murine blood recorded after infection with *K. pneumoniae* YJH4 and YJH15 for 24 hours by quantitative real-time PCR (n = 4). Mice injected with normal saline were included as normal control. Data were presented as the mean ± SD of four independent cDNA samples and analyzed by the unpaired *t* test (***P = 0.0007).

The expression levels of blood inflammatory cytokines in ICR mice infected with strain YJH4 and YJH15 were determined and compared to those of non-infected mice. The expression levels of cytokines IL-1β, IL-10 and IFN-γ were found to increase in infected mice, whereas that of cytokines IL-6, IL-12b and TNF-α were lower in the blood of infected mice, when compared to the non-infected control (**Figure 3**). In addition, colistin-resistant strain YJH15 induced similar levels of all tested cytokines as colistin-susceptible parental strain YJH4, except that the expression level of IL-1β induced by strain YJH15 was significantly lower than that induced by strain YJH4 (*P* = 0.0007). These data suggested that infection of mice by *K. pneumoniae* is accompanied with a robust immune response among which infection of strain YJH15 triggered less IL-1β expression than strain YJH4, but the expression level of IL-1β was still much higher than that in non-infected mice.

## Discussion

Multidrug-resistant *K. pneumoniae* is one of the most common causative agents of nosocomial infections including sepsis, pneumonia, meningitis and urinary tract infections. It is also one of the most common carbapenem-resistant pathogens isolated in healthcare settings (Sugden et al., 2016; Sherry and Howden, 2018). In the past few decades, polymyxins including polymyxin B and colistin have been the most commonly used antibiotics for treatment of infections caused by carbapenem-resistant *K. pneumoniae*; these agents are therefore regarded as the last resort antimicrobial options for treatment of infections caused by multidrug-resistant *K. pneumoniae* strains. However, with a sharp increase in usage of polymyxins in agriculture and healthcare settings in recent decades, colistin-resistant *K. pneumoniae* strains have emerged in recent years. The proportion of colistin resistance among carbapenemase-producing *K. pneumoniae* has also gradually increased from less than 2% worldwide in the last decade to 9% in recent years (Gales et al., 2011; Gupta et al., 2011; Marchaim et al., 2011; Mezzatesta et al., 2011). Recent studies have also revealed a set of concerning data in several European regions, in which the rate of resistance to colistin among clinical carbapenem-resistant *K. pneumoniae* has risen to 43% in Italy, 31% in Spain and up to 20.8% in Greece (Monaco et al., 2014; Pena et al., 2014; Meletis et al., 2015). In this study, we compared the relative fitness and virulence level in colistin-susceptible and colistin-resistant *K. pneumoniae* strains that emerged as a result of *mgrB* gene inactivation, using both *in vitro* and *in vivo* models. The present study utilized our unique materials, a colistin-resistant clinical *K. pneumoniae* strain YJH15, which was selected from a colistin susceptible strain YJH4 in a patient due to the use of colistin as treatment and contained chromosomal *mgrB* gene mutations. The use of a clinically evolved colistin-resistant *K. pneumoniae* strain on assessing the virulence potential might be more relevant to the clinical situation.

Fitness and virulence evolution caused by colistin resistance-inducing chromosomal mutations have previously been investigated mostly in *K. pneumoniae* and *Acinetobacter baumannii*. These previous studies reported impaired growth and decreased virulence in colistin-resistant *A. baumannii* (López-Rojas et al., 2011; López-Rojas et al., 2013; Pournaras et al., 2014). In addition, reduced fitness and virulence due to *mcr-1* bearing plasmid-mediated colistin resistance has been reported in *Escherichia coli* (Tietgen et al., 2018). Furthermore, it was also demonstrated that fitness cost associated with an *mcr-1*-bearing plasmid could be progressively diminished in serial cultures (Ma et al., 2018). A previous study also revealed that carriage of *mcr-1*-bearing plasmid in *K. pneumoniae* was associated with a fitness cost (Nang et al., 2018). However, not all mechanisms of colistin resistance impaired physiological fitness in the bacterial host; for instance, Cannatelli et al. reported that development of colistin resistance due to chromosomal deletion of *mgrB* in *K. pneumoniae* did not have significant fitness cost (Cannatelli et al., 2015). Similarly, the fitness and virulence potential of YJH4 and colistin-resistant mutant YJH15 were different in different assays and models in this study. The growth rates of these two strains were similar, while YJH15 actually exhibited increased fitness in *in vitro* competition experiments. Most importantly, virulence potential of YJH15 was significantly lower than that of YJH4. These data indicated even though the *in vitro* assays and *G. mellonella* data showed that YJH15 exhibited higher fitness after becoming colistin-resistant, its virulence actually reduced dramatically in competition and virulence assay in mice model, which should reflect the virulence potential of *K. pneumoniae* in human. In addition, colistin-resistant *K. pneumoniae* YJH15 exhibited decreased virulence in mouse infection model but increased virulence in *G. mellonella*, such discrepancy was also reported by Russo TA et al. (Russo and MacDonald, 2020) in which the outbred mouse infection model was highly accurate for differentiating the virulence of hypervirulent *K. pneumoniae* from classical *K. pneumoniae* strain, while a significant overlap in virulence was observed in the *G. mellonella* infection model. And they suggested that strains in which the pathogenic potential is ambiguous due to the antimicrobial resistance that could decrease fitness should be validated in an outbred murine model. Increased virulence of colistin-resistant strain in *G. mellonella* infection model might be partially due to cross-resistance to antimicrobial peptides naturally produced by *G. mellonella* larvae as part of their innate immunity (Brown et al., 2009). Similar results were also observed in Gerson *et al*’s study in which colistin-resistant *A. baumannii* strains due to *pmrB* and *eptA* mutations exhibited increased virulence in *G. mellonella* infection model when compared with their colistin-susceptible isogenic strains (Gerson et al., 2019). IL-1β is a potent inflammatory cytokine that induces the expression of adhesion molecules and regulates the influx of inflammatory cells (Dinarello, 2005). The cytokine is also strongly involved in adaptative immunity and production of IL-1β is firmly mediated at both transcriptional and post-translational levels. Lipid A modifications can affect the stimulatory properties of lipopolysaccharides (LPS) and recognition of bacterial cells by innate immunity. For example, the modification of pET to lipid A decreases the autophagy process activation in human macrophages (Zughaier et al., 2014), suggesting that it could be a mechanism to escape the host immune system. It was also reported that the expression of *mcr-1* gene in *E. coli* resulted in an immunomodulatory phenotype with a decreased expression of genes for the proinflammatory cytokines TNF-α and IL-1β (Mattiuz et al., 2020). Kamoshida et al. reported that colistin-resistant *Acinetobacter baumannii* due to a deficient LPS induced lower expression levels of IL-1β, IL-6 and IL-8 in neutrophils when compared with its colistin-susceptible wild type (Kamoshida et al., 2020). LPS are important factors underlying the capability of bacteria to resist serum-mediated bactericidal activity by the host (Ciurana and Tomás, 1987), hence modification of LPS in colistin resistant strains will compromise the ability to counteract host defence. A previous work also reported that *K. pneumoniae* lipopolysaccharide and capsule polysaccharide were responsible for conferring resistance to neutrophil phagocytosis *in vitro* and killing by serum (Tomás et al., 1986).

Emergence of colistin and carbapenem resistant *K. pneumoniae* strains is a major public health concern. This study delineated the impact of *mgrB* inactivation-induced colistin resistance on the physiological fitness and virulence of carbapenemase-producing *K. pneumoniae* strains and found that colistin resistance was associated with reduced fitness and virulence *in vivo*, but enhanced fitness *in vitro*. The enhanced fitness in colistin-resistant strains *in vitro*, along with the high utilization rate of polymyxins in both healthcare settings and agriculture, may impose strong selection pressure that facilitate rapid dissemination of colistin-resistant organisms in the environment. It is necessary to stress that findings in this work suggest that *mgrB* gene inactivation is associated with a variety of changes in fitness and virulence related phenotypes, yet the underlying molecular mechanisms that cause these phenotypic changes remain unknown. Further study will focus more on the mechanisms of such phenotypic changes caused by evolved colistin resistance in *K. pneumoniae*.

## Data Availability Statement

The raw data supporting the conclusions of this article will be made available by the authors, without undue reservation.

## Ethics Statement

The animal study was reviewed and approved by Research Animal Care and Use Committee of City University of Hong Kong.

## Author Contributions

MX performed the experiments and drafted the manuscript. KC helped with animal experiments. ND helped with genome sequencing. QX performed cell killing assay. EC edited the manuscript. RZ was responsible for collection of clinical strains and data. SC supervised the project and edited the manuscript. All authors contributed to the article and approved the submitted version.

## Funding

This study was funded by Guangdong Major Project of Basic and Applied Basic Research (2020B0301030005).

## Conflict of Interest

The authors declare that the research was conducted in the absence of any commercial or financial relationships that could be construed as a potential conflict of interest.

## Publisher’s Note

All claims expressed in this article are solely those of the authors and do not necessarily represent those of their affiliated organizations, or those of the publisher, the editors and the reviewers. Any product that may be evaluated in this article, or claim that may be made by its manufacturer, is not guaranteed or endorsed by the publisher.
